# *In Vitro* Liver Models for Studying Pharmaceutical Metabolism in Fish – A Critical Analysis of Their Applications and Limitations

**DOI:** 10.1021/acs.est.6c00280

**Published:** 2026-05-27

**Authors:** Chrisna Matthee, Tea L. M. Pihlaja, Päivi Järvinen, A. Ross Brown, Anke Lange, Tiina M. Sikanen, Charles R. Tyler

**Affiliations:** † Faculty of Health and Life Sciences, Biosciences, 3286 University of Exeter, Exeter EX4 4QD, United Kingdom; ‡ Faculty of Pharmacy, Drug Research Program, 3835 University of Helsinki, Helsinki FI-00790, Finland; § Helsinki Institute of Sustainability Science, University of Helsinki, Helsinki FI-00100, Finland

**Keywords:** bioaccumulation, biotransformation, ecotoxicology, environmental risk assessment, nonanimal methods

## Abstract

Bioaccumulation studies performed as part of the environmental risk assessment of human pharmaceuticals are time- and resource-intensive, prompting interest in alternative nonanimal screening methods. For xenobiotics, including active pharmaceutical ingredients (APIs), biotransformation and metabolic clearance are key determinants of bioaccumulation and, consequently, effects in nontarget organisms like fish. Various *in vitro* liver models are available for studying pharmaceutical metabolism in fish, including microsomes, S9 fractions, and primary hepatocyte cultures maintained as suspensions, monolayers, or three-dimensional spheroids. However, broader application of these models is limited by a lack of robust *in vitro-in vivo* correlation data, as well as uncertainty regarding the most appropriate systems for different modeling purposes. This study critically evaluates *in vitro* liver models commonly used for assessing API clearance in fish, comparing their functionality, resource requirements, and informative value. Drawing on published data for selected APIs and on the strategies used to select suitable mammalian model systems, we propose the use of subcellular fractions for high-throughput screening of hepatic clearance (metabolic stability) and enzyme inhibition, hepatocyte suspensions for assessing intrinsic clearance while accounting for the impact of active drug transport, and hepatocyte monolayers or three-dimensional cultures for longer-term clearance and targeted effect studies. Key knowledge gaps are identified, and recommendations for future research are presented.

## Graphical abstract

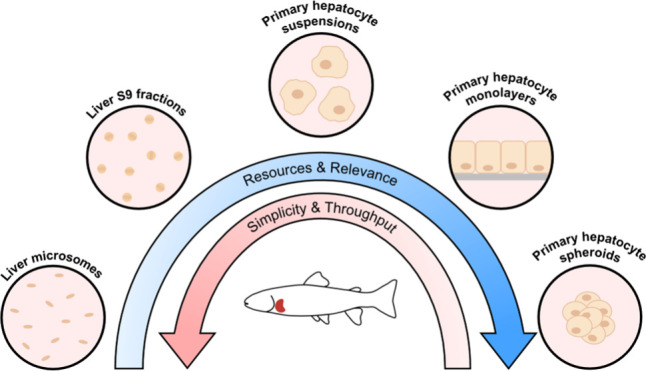

## Introduction

As a result of the global rise in medicine usage, active pharmaceutical ingredients (APIs) and their metabolites are increasingly detected in the environment, particularly in aquatic systems, where they may pose a risk to sensitive nontarget organisms. Although levels of APIs detected in water bodies (typically in the low ng/L to low μg/L range)^1,2^ are generally not expected to cause significant harm, physiologically active concentrations have been found in exposed wildlife and have, in some cases, been causally linked to adverse effects on individual health and fitness.^3–6^ Compared to many other wildlife species, fish are at relatively high risk of exposure to pharmaceuticals as they often live in surface waters receiving high level inputs of municipal and industrial waste streams. The vulnerability of fish to drug exposure is also greatly influenced by their distinct physiological features that underly drug pharmacokinetic processes, including metabolism, as reviewed by Matthee et al.^7^ Since 2006, the European Union has mandated that all human medicinal products undergo an environmental risk assessment (ERA) to evaluate their potential ecological impacts prior to the granting of marketing authorization.^8^ As part of this assessment process, conducted in accordance with guidance issued by the European Medicines Agency (EMA),^9^ compounds that exceed established lipophilicity thresholds are evaluated *in vivo* for their bioaccumulation potential in fish. Assuming the main route of uptake into the body is from water (via the gills), bioaccumulation is quantified using either a steady-state or kinetic bioconcentration factor (BCF), with a BCF > 2 000 as the threshold for bioaccumulative substances.^9^ These tests are time-consuming, costly and require large numbers of fish to be sacrificed.^10^ Additionally, most of the pharmaceuticals tested to date have not been found to be bioaccumulative as defined by the EMA guideline.^11,12^ Given the multitude of legacy pharmaceuticals (authorized prior to 2006 without a formal ERA) and the annual approval of up to 60 new products,^13^ testing the bioaccumulation potential of every API that meets the set lipophilicity criteria is both unethical and impractical.

Drug metabolism in humans is studied at various stages of the drug discovery and development process as it plays a central role in determining both the therapeutic efficacy and toxicity profile of a compound. During such studies, a range of mammalian *in vitro* liver models – ranging from subcellular fractions to whole-cell systems – are routinely employed to predict metabolic liabilities, assess metabolic stability, and identify metabolites, metabolic pathways and metabolism-related interactions (Figure 1). This is based on the knowledge that the liver expresses high levels of drug-metabolizing enzymes (DMEs; Supporting Information, Table S3) and is the main site of drug metabolism in humans, although extrahepatic metabolism can also occur in organs such as the gut and kidneys.^14^ In this context, these *in vitro* tests aim to ensure that the drug can be safely and effectively administered and tested in humans. Similarly, in nontarget organisms such as fish, hepatic metabolism is often a major determinant of xenobiotic clearance and bioaccumulation potential.^15^ Various *in vitro* liver models – developed using approaches similar to those for mammals – are available for studying pharmaceutical metabolism in fish, but systematic comparison of data from different fish models is lacking, which hinders the use of *in vitro* fish data in the context of ERA.

**Figure 1:**
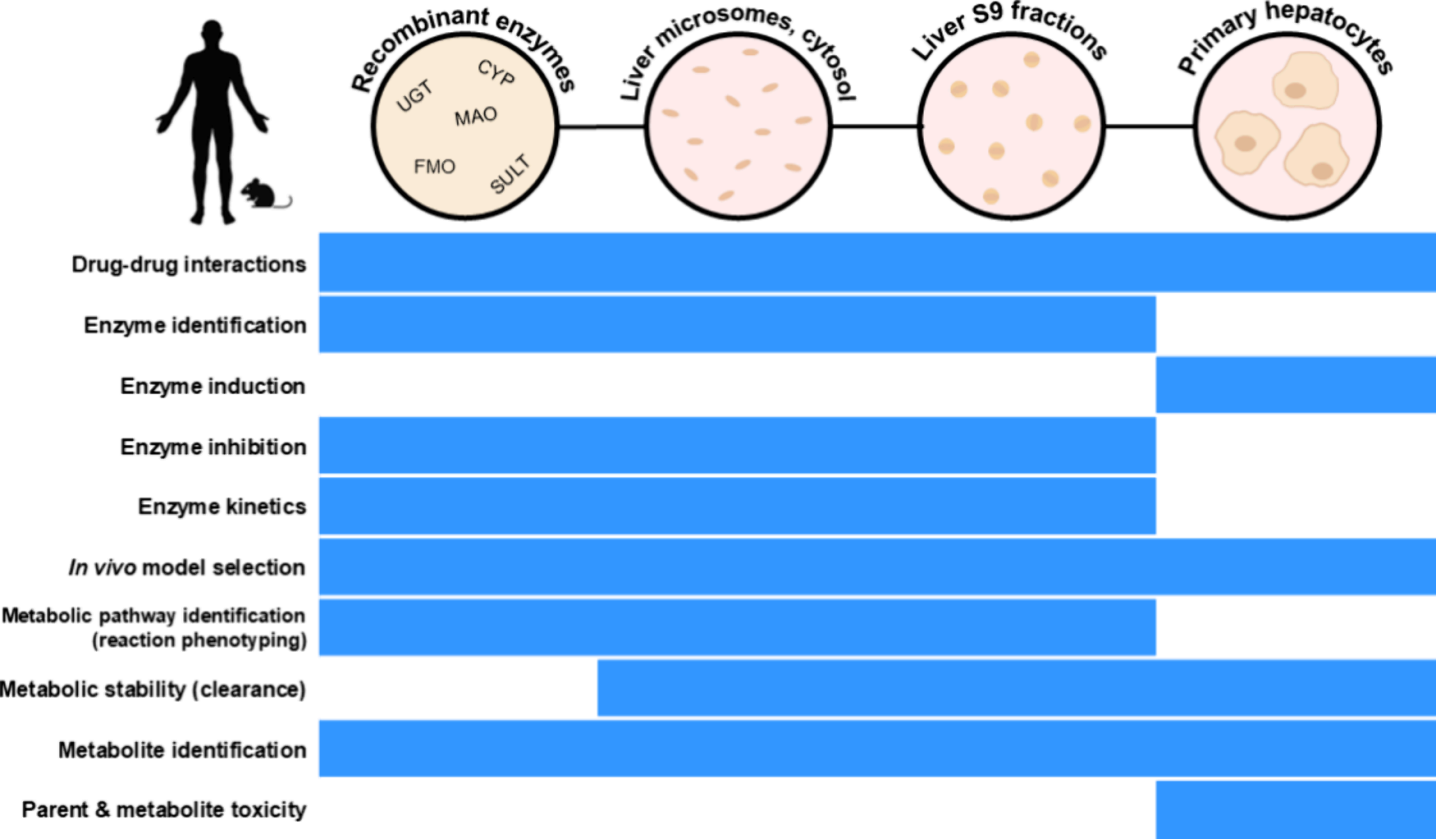
Applications of mammalian *in vitro* liver models during the drug discovery and development process. Blue and white cells indicate whether the model is commonly applied for the indicated purpose or not, respectively. This classification reflects practical applicability rather than the full range of theoretical capabilities. Based on literature.^16–19^

In mammalian studies, primary hepatocytes isolated from the liver and used directly for cell culturing and testing are often regarded as the gold standard for the *in vitro-in vivo* extrapolation of drug metabolism.^20^ These cells can be cultured in suspension or as monolayers (i.e., attached to an artificial growth substrate). In addition, primary hepatocytes can be used to establish three-dimensional (3D) spheroid cultures. The available subcellular systems, on the other hand, include liver microsomes as well as cytosolic and S9 fractions. Microsomes are small, sealed vesicles that contain the contents of the endoplasmic reticulum. These artifacts are not present in living cells but form from fragmented cell membranes during cell homogenization and can be separated from the cytosolic fraction by differential centrifugation.^21^ The S9 fraction is also obtained from the liver homogenate but contains both the microsomes and cytosolic fraction of the cell. Several recombinant enzyme systems have also been developed in which specific human metabolic enzymes are expressed in *in vitro* systems lacking endogenous human DME-encoding genes, such as bacterial cells (e.g., *Escherichia coli*), as well as insect- (e.g., baculovirus-infected *Spodoptera frugiperda* Sf9) and mammalian cell lines (e.g., human embryonic kidney HEK293).^22^ Currently, the majority of drug metabolism assays, including both cell-based and subcellular assays, are performed in a well-plate format, which is compatible with high-throughput screening. Substantial research efforts, however, have more recently focused on developing microfluidic (flow-through) assay systems aimed at enhancing cell viability and morphological maturation (a.k.a. organ-on-a-chip)^23^ or enabling mechanism-based drug interaction studies with subcellular fractions,^24^ either as standalone platforms or in combination with multiorgan-on-a-chip configurations.^25^

To assess pharmaceuticals elimination in fish, the standard approach is to determine the BCF. Currently available *in silico* tools used to predict fish BCFs, however, often fail to adequately account for metabolic biotransformation, which contributes to their only moderate predictive performance.^26^ In this regard, *in vitro* and *ex vivo* fish liver models represent useful screening tools that can be employed during ERA to better understand and infer (i) compound bioavailability (i.e., the amount of unchanged chemical available to elicit a biological response), (ii) bioconcentration and/or bioaccumulation potential, and (iii) drug metabolic profiles (metabolites, metabolic pathways and metabolism-associated interactions) in fish, providing additional weight of evidence alongside *in silico* models to evaluate the need for *in vivo* fish testing.^27^ However, standardized methods are currently available only for the determination of intrinsic clearance (CL_INT_) *in vitro* using rainbow trout S9 fractions (OECD Test Guideline 319B)^28^ and primary hepatocyte suspensions (OECD Test Guideline 319A),^29^ as well as for the extrapolation of *in vitro* data from these systems to *in vivo* CL_INT_ and its incorporation into BCF calculations.^30^ However, careful evaluation of the most suitable *in vitro* model for specific contexts is essential to enable focused and coordinated validation efforts in pharmaceutical ERA.

In this review, we critically evaluate the different *in vitro* fish liver models that are, at present, most commonly used to study pharmaceutical metabolism in fish (Figure 2). These include fish liver microsomes, liver S9 fractions and different types of primary hepatocyte cultures. Liver spheroid cultures are included in this analysis as these models are increasingly being developed and evaluated for their feasibility in fish xenobiotic metabolism studies, especially for environmental applications.^31^ It is worth noting, however, that mammalian liver spheroid cultures were originally developed mainly to study liver function, liver disease progression, and drug-induced liver injury,^32^ and have only more recently been adapted to investigate pharmaceuticals with low hepatic clearance.^33^ Immortalised liver cell lines, on the other hand, are not a focus of this analysis. While certain fish cell lines have been shown to possess diverse metabolic enzyme functionality,^34^ their overall metabolic capacity is not especially well quantified and thus their physiological relevance for metabolism studies is generally considered inferior to that of other liver models.^35–37^ This limitation largely stems from cell lines’ dedifferentiated state, which often implicates a reduced expression of DMEs. These cells may lose their phenotypic characteristics,^38^ further diminishing enzyme activity and introducing additional uncertainty when performing *in vitro-in vivo* extrapolations or drawing comparisons with other *in vitro* systems containing intrinsic DMEs. Microfluidic assay systems are also not included in our analyses as their application in fish research is currently limited^39,40^ and insufficiently characterized. The model systems selected (Figure 2) are compared in terms of their functionality, resource requirements and capacity to provide additional information, highlighting the strengths and limitations of each system (summarized in Supporting Information, Table S4). This is done, in turn, to identify the most suitable *in vitro* model for a range of applications – particularly as pharmaceutical ERA screening tools – and deduce whether model selection can be usefully informed by current mammalian *in vitro* approaches.

**Figure 2:**
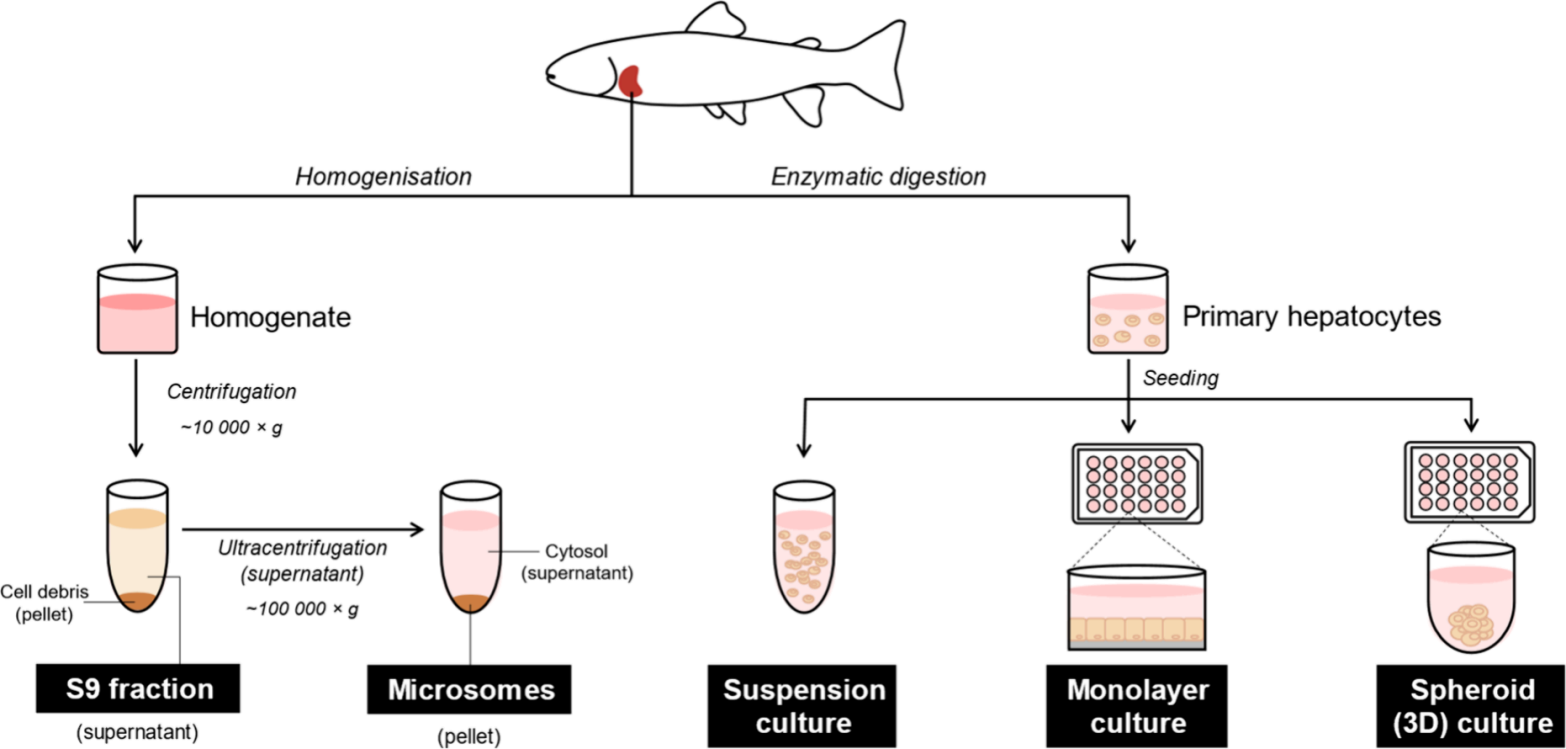
Overview of *in vitro* liver subcellular and cellular culture systems commonly used in pharmaceutical metabolism studies in fish, including an outline of their origin and development.

In addition to the present review, the reader is referred to previous reviews discussing *in vitro* fish liver systems from alternative viewpoints.^41,42^ These previous reviews generally survey the full range of available systems (including several that are rarely used for CL_INT_ determinations), summarizing technical details and assessing the integration of *in vitro* data into bioaccumulation models and regulatory assessments. For a comprehensive overview of biotransformation processes and systems in fish, we also refer the reader to the latest edition of Toxicology of Fishes.^43^ Whereas previous reviews have centered on pesticides, industrial chemicals, or broadly defined ‘commercial substances’, this review focuses specifically on APIs, which are generally designed to be biologically active at low concentrations and target human pathways that are widely conserved in fish.^44,45^

## Critical Analysis of *in Vitro* Fish Liver Models

### Functionality and Performance

#### Physiological Relevance

Liver S9 fractions incorporate the majority of both phase I enzymes and phase II transferases (Supporting Information, Table S3), offering a relatively comprehensive metabolic profile that is arguably comparable to that of whole-cell (and even whole-animal) systems. Liver microsomes, on the other hand, include only the membrane-bound enzymes of the endoplasmic reticulum – such as the cytochrome P450s (CYPs) and uridine 5′-diphospho-glucuronosyltransferases (UGTs) – making it a more limited but still relevant model as these enzymes are responsible for the majority (>80%) of metabolic reactions and associated interactions of pharmaceuticals in humans.^20,46,47^ Neither of these subcellular systems, however, can account for the impacts of drug transport across the cell membrane, nor do they possess the machinery needed for the expression of new functionally active DMEs. Moreover, both these systems have a limited functional lifespan, with enzyme activity typically being lost within a few hours, and they require the addition of cellular cofactors during *in vitro* assays to enable specific metabolic reactions (Supporting Information, Table S3).

Compared with subcellular fractions, primary hepatocytes more closely represent *in vivo* conditions as they contain all the relevant cell organelles, metabolic enzymes and membrane transporters. Moreover, hepatocytes possess the necessary molecular machinery for encoding these proteins, which enables the study of their inducibility. While freshly isolated hepatocytes are generally preferred due to their superior retention of native cellular functionalities, cryopreserved hepatocyte suspensions have also been successfully applied in CL_INT_ determinations,^48^ offering a practical and accessible alternative that facilitates broader applicability and reproducibility in experimental workflows. A key limitation in fish primary hepatocyte research, however, is the lack of a standardized hepatocyte isolation protocol across species, which may introduce substantial variability that, in turn, hinders both interlaboratory comparison and method validation. Currently, robust protocols are available mainly for rainbow trout^29,49^ and common carp,^50^ thereby limiting efforts to account for interspecies differences. Suspension cultures of primary hepatocytes are relatively simple to prepare and, when maintained under shaking conditions, the formation of diffusion gradients that may limit oxygen and nutrient supply to the cells can be avoided. However, it is well-known that primary hepatocytes in suspension lose their differentiated state within a relatively short time period (i.e., a few hours) due to the lack of cell–cell interactions. This process can be slowed by culturing the cells in 2D or 3D configurations. When cultured as monolayers, primary fish hepatocytes have been shown to rapidly recover from isolation and seeding-induced stress, and to retain most *in vivo*-like structural and functional characteristics for several days.^51,52^ The re-establishment of cell–cell contacts achieved during monolayer formation is critical in this regard.^53^ The static incubation conditions used for the monolayer system, however, contrast with the dynamic blood supply present *in vivo*.^54^ A further limitation of both primary hepatocyte monolayers and suspensions is that these systems lack cellular heterogeneity and generally use a lower cell density compared with the intact liver.

Research into 3D cell culture techniques, including the use of hepatocyte spheroids (also known as aggregate cultures), has surged over the past decade. A general advantage of these systems over conventional 2D liver models is that they enable cells to establish polarity and form intercellular junctions, which are essential for performing some of the liver’s more complex functions.^55,56^ Primary human hepatocyte spheroids, for example, have been shown to maintain hepatocyte functionality and morphology, as well as a proteomic profile that closely resembles that of the *in vivo* human liver, over prolonged culture periods.^33^ The majority of data available on 3D liver cultures, however, pertain to human hepatocytes, as they are routinely used in drug discovery. Nonetheless, the superior metabolic capacity of fish hepatocyte spheroids over simpler *in vitro* models was already demonstrated in the 1990s.^57,58^ The use of 3D fish hepatocyte cultures enables longer-term exposure experiments, given that spheroids often maintain good viability, functionality and cellular differentiation for up to 30 days (or more).^59,60^ Furthermore, gene expression levels in fish liver spheroids during later stages of culture have been reported to more accurately represent those *in vivo*, compared with freshly isolated hepatocytes and monolayer cultures.^61^ In addition to primary fish hepatocytes, immortalised cell lines such as RTL-W1 and RTH-149 have also been used in the development of 3D liver models.^37,62^ This approach shows promise for redifferentiating the cells to a hepatocyte-like phenotype, including the upregulation of metabolism-associated genes, and appears more effective than conventional 2D models.^63^ The increased complexity of 3D liver models, however, comes with caveats and limitations. For example, the high variability associated with spheroids in terms of their size and shape in culture, which affect cell viability, metabolic capacity and, ultimately, experimental reproducibility, has often been a point of discussion.^62^ Accordingly, several 3D well plate formats have been developed to control for spheroid size, aiming to form either a single large spheroid^37^ or multiple smaller, uniformly sized spheroids in each culture well.^64^ While reproducibility relating to uniform-sized spheroids within a given assay setup is generally no longer a major concern, comparing results across different 3D culture systems is still challenging. This is due to substantial biological differences that occur for spheroids of varying diameters (e.g., 500 μm versus 50 μm), arising from factors such as zonation, cell density, and the transport of oxygen, nutrients and APIs to the spheroid core.^65^ The diversity among 3D liver models and their applications (e.g., *in vitro* cytotoxicity versus intrinsic clearance determinations) ultimately complicates the selection of a best-fit model for standardizing 3D culture approaches.

#### Hepatic Clearance

The extent of pharmaceutical metabolism is typically expressed with the help of a measure of *in vitro* intrinsic clearance (CL_INT_; mL/min per cell count or mg protein), which estimates the ability of the liver to remove the drug in the absence of flow limitations and binding to cells and/or proteins in the blood.^66^ Using appropriate scaling factors and extrapolation models (such as the well-stirred liver model, parallel tube model or dispersion model),^30,67^ the hepatic CL_INT_ determined *in vitro* can be extrapolated to *in vivo* hepatic clearance, which quantifies the volume of blood cleared of API by the liver per unit time (typically in mL/min or L/h).^68^ A number of studies have compared *in vivo* clearance estimates obtained from different mammalian *in vitro* liver systems. While some have found hepatocytes to be superior to subcellular fractions for these predictions,^69,70^ others have shown that whole-cell and subcellular systems generally produce results that are concordant.^71^ Predicted clearance values obtained from fish hepatocytes and subcellular fractions have also been found to be in strong agreement.^72,73^ When comparing the extrapolated *in vivo* CL_INT_ (mL/h/g liver) of five selected APIs across four different rainbow trout *in vitro* liver models (Figure 3),^64,74,75^ variation among the results is evident and is likely attributable to both assay-specific differences and to the fact that S9 fractions and cells were often derived from multiple individual fish (only primary cells used in suspension and spheroid assays originated from the same fish). Nevertheless, S9 fractions and monolayer cultures showed similar trends in terms of clearance efficiency (i.e., propranolol > mycophenolic acid (MPA) > quetiapine > clozapine ∼ olaparib), although clearance of clozapine and olaparib were only detectable in the monolayers, but not in the S9 fractions. In the hepatocyte suspensions, clearance of quetiapine and olaparib were notably higher compared with other *in vitro* models, whereas considerable variation was associated with the clearance determinations performed using the spheroid cultures. Interestingly, no clearance of quetiapine was evident in the hepatocyte spheroids, while all the other *in vitro* models showed measurable clearance of this API. These differences in API clearance among the models may reflect interindividual variation among donor fish, as well as differences in available metabolic pathways resulting from variable expression of functionally active DMEs (in cultured hepatocytes).^75^ On the other hand, the *in vitro*-derived clearance of propranolol in rainbow trout liver S9 fractions reported by others (137 mL/h/g liver)^76^ falls within the variation observed in these studies (Figure 3), evidencing the reproducibility of *in vitro* assay outcomes independent of the model used.

**Figure 3:**
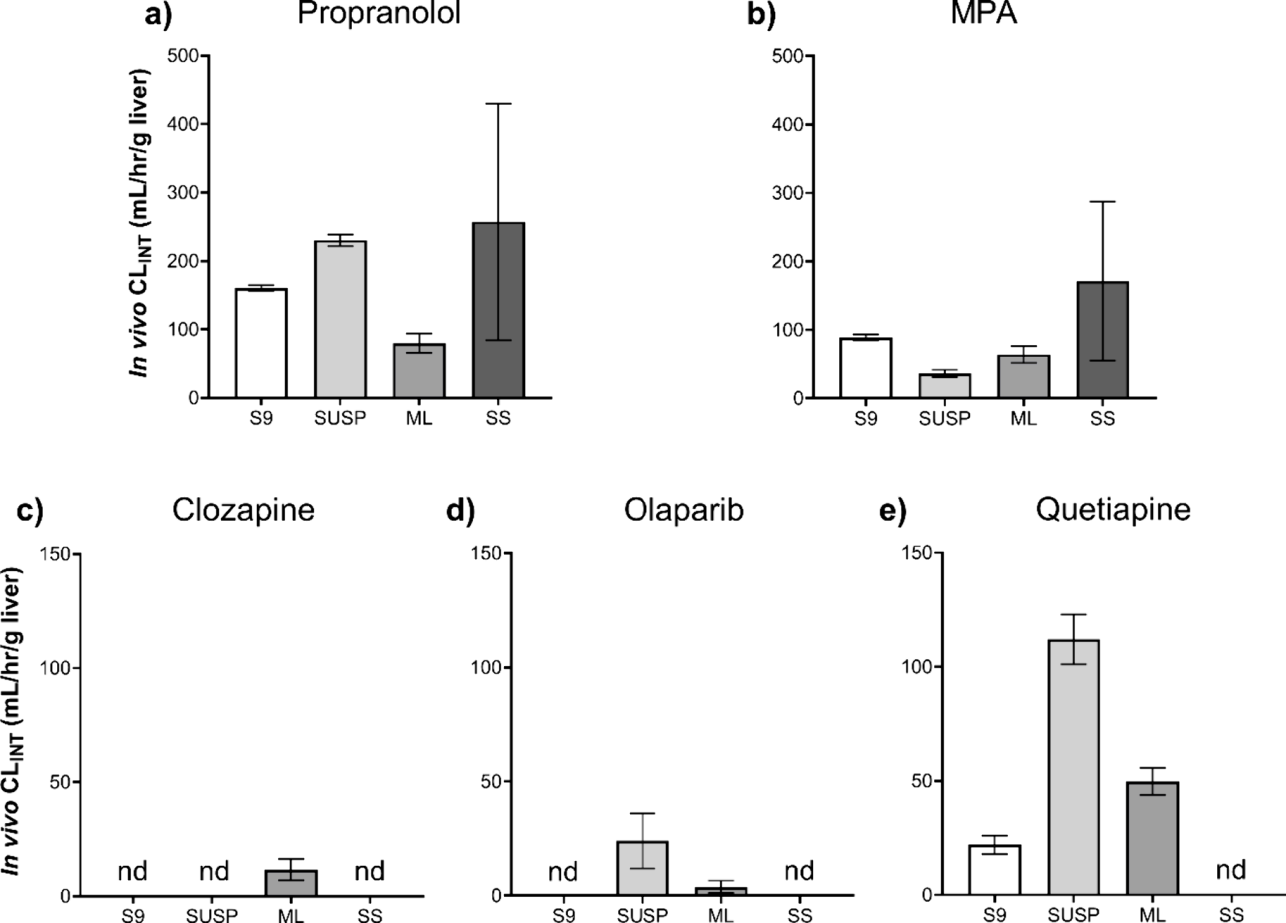
Extrapolated *in vivo* intrinsic clearance (CL_INT_) values for selected active pharmaceutical ingredients across different rainbow trout *in vitro* liver models. Selected active pharmaceutical ingredients include propranolol (a), MPA (b), clozapine (c), olaparib (d) and quetiapine (e). Clearance values were extrapolated from *in vitro* CL_INT_ data and are depicted as the mean ± standard error of the mean. A nominal exposure concentration of 1 μM was used in all cases. Olaparib clearance values in SUSP and SS are derived from authors’ unpublished data (see Supporting Information for experimental details and meta-data). All other data are based on literature.^64,74,75^ Abbreviations: ML, primary hepatocyte monolayer cultures; MPA, mycophenolic acid; nd, clearance not detectable; S9, liver S9 fractions; SS, static primary hepatocyte spheroid cultures; SUSP; primary hepatocyte suspension cultures.

When considering the precision of clearance measurements for each of the selected model systems (as indicated by the standard error in the data, Figure 3), S9 fractions appear to produce the most consistent results, followed by the hepatocyte (suspension and monolayer) models and then the spheroids. Despite substantial evidence demonstrating good reproducibility across the different *in vitro* systems, their accuracy in predicting *in vivo* outcomes remains insufficiently characterized. Direct comparison of *in vivo* CL_INT_ extrapolations with measured elimination half-lives in fish is typically not feasible, unlike the routine monitoring of API elimination in human plasma. Instead, correlations must be established by comparing measured steady-state BCFs with those predicted from *in vitro* data. Efforts have however been made to examine the *in vitro-in vivo* translatability of fish primary hepatocyte suspensions and liver subcellular fractions. These studies have largely demonstrated qualitative concordance, such as the ability to predict the occurrence of clearance,^77^ or identifying relevant metabolic pathways and metabolites.^42^ The few studies that have applied a quantitative approach have used either isolated perfused fish livers (i.e., *ex vivo*) to obtain representative *in vivo* clearance values^78,79^ or retrospectively derived these values from other clearance measurements.^80^ Notably, however, these studies primarily focused on organic, nonionizable environmental pollutants, rather than on APIs.

#### Metabolic Enzyme Activity

The metabolic capacity of *in vitro* liver models is commonly assessed by studying the relative activity of their phase I (i.e., CYP and carboxyl esterase) and phase II (i.e., glutathione transferase and UGT) metabolic enzymes.^29^ The average basal activities of the main enzymes involved in API metabolism differ widely across different *in vitro* fish liver models (Supporting Information, Table S5). This variability needs to be carefully considered when comparing these models and selecting the most appropriate one for a specific application. Additionally, the source of hepatocytes plays a key role: using cells from a single donor fish enables the investigation of interindividual variability, whereas the use of pooled subcellular fractions or hepatocytes from multiple fish helps to reduce variability, making the latter more suitable for high-throughput screening approaches. On average, liver subcellular fractions show higher protein-normalized phase II (UGT) enzyme activity (see Supporting Information, Table S5). This is reflected in the comparative clearances of MPA (Figure 3b), which primarily undergoes glucuronidation in humans. Phase I enzyme activities, on the other hand, seem to be relatively comparable across the different cellular and subcellular models (see Supporting Information, Table S5). Comparisons across and within the different *in vitro* systems, however, may be hindered by substantial methodological variability among studies. These differences may include variations in cell viability and protein quantification methods, donor fish strain and age, and the choice of reaction substrates. Furthermore, even when the same marker reaction is employed, factors such as substrate concentration and other experimental conditions (e.g., buffer composition, protein/cell concentration) can affect results, emphasizing the importance of establishing standardized assay conditions.

### Resource Requirements

In addition to performance and functionality, the costs involved, time required and complexity of the experimental setup for each *in vitro* system need due consideration when selecting the most appropriate model for any given application. Comparisons between the use of human hepatic S9 fractions and hepatocytes in metabolic stability assays have shown that the hepatocyte assay is over 20 times more expensive.^71^ Similar cost differences are likely across *in vitro* fish liver models, with 3D spheroid cultures being the most expensive due to the need for specialized consumables. Overall, cell-based assays are estimated to be approximately 15 times more costly than subcellular fraction assays (see Supporting Information, Table S6). Regardless of the *in vitro* model being employed, cost considerations include fish husbandry when performing in-house primary hepatocyte isolations, or the acquisition of cryopreserved subcellular fractions or hepatocytes. Notably, the cost of cell-based assays depends heavily on seeding density and the hepatocyte source. Primary fish hepatocytes are generally readily accessible, and the availability of cryopreserved cells now also offers greater flexibility in terms of experimental planning. Despite the need for additional cofactors and pore-forming agents (to overcome the latency effect in kinetic determinations), fish liver subcellular systems remain substantially more affordable than cell-based models. By contrast, monolayers and 3D cultures often require additional media supplements to maintain cell viability for prolonged culture periods, as well as specialized culture plates and other equipment.

A similar trend can be seen when comparing the time required to set up and carry out API clearance assays with these models (Supporting Information, Table S6). For subcellular fractions, assay preparation is relatively simple and exposure experiments – during which multiple APIs can be tested simultaneously – only take up to 4 h. In comparison, while hepatocyte suspension cultures require around the same time as subcellular fractions, monolayers and spheroid cultures require additional time-consuming steps for cell seeding and culturing. Rainbow trout primary hepatocyte spheroids, for example, have been shown to take up to 10 days to reach functional and morphological ‘maturity’ ^31,61^ – a prerequisite for their use in further ecotoxicological assessment. Moreover, the technical demands of the monolayers and (particularly) spheroid cultures – such as the need for surface coatings, accurate cell counting, careful handling, culture medium changes and microscopic examination – restrict the number of APIs that can be tested in parallel. In turn, however, the prolonged exposure periods, combined with the enhanced biological complexity of these systems, offer additional advantages. These include achieving a more physiologically relevant phenotype and enabling longer-term exposures, thereby facilitating clearance determinations of slowly metabolized APIs, as well as enzyme induction and cytotoxicity studies (Supporting Information, Table S4). Analytical method development and validation for quantifying substrate depletion is generally required across all the *in vitro* assay types, although minor variations may arise due to differences in sample preparation requirements between matrices.

It should be noted that the cost and time considerations discussed above are based on the authors’ experience and on limited literature, both of which focus almost exclusively on the rainbow trout. These estimates may differ across species, however, highlighting the need for expanded efforts to characterize possible interspecies differences.

### Informative Value

#### Estimating Bioconcentration Factors and Elimination Half-Lives

During pharmaceutical ERA, *in vivo* fish bioaccumulation tests are only warranted for compounds with *n*-octanol–water partition coefficients (log K_OW_) > 4.5, or for compounds with log K_OW_ ≥ 3 and a predicted environmental concentration in surface water ≥ 0.01 μg/L.^9^ As a result, empirically determined fish BCFs are lacking for many APIs (particularly those approved prior to the introduction of mandatory ERA). Moreover, due to inherent differences in pharmaceutical exposure profiles, as well as in absorption, metabolism and excretion routes among taxonomic groups,^7^ measures of clearance in mammals are unlikely to be useful for predicting drug bioaccumulation in fish. The integration of *in vitro* clearance data from both fish hepatocytes and liver subcellular fractions to refine *in silico*-predicted BCFs has demonstrated considerable promise in this regard^73,81–86^ – thereby evidencing some *in vitro-in vivo* translatability – and a standardized method for applying *in vitro-in vivo* extrapolation to estimate BCFs from *in vitro* clearance data already exists for rainbow trout primary hepatocyte suspensions and liver S9 fractions.^30^ However, the applicability of these approaches to ionizable compounds – such as most APIs – remains uncertain and requires further validation, particularly regarding their *in vitro-in vivo* correlation for BCF prediction.^87^ More specifically, when following the Organisation for Economic Cooperation and Development (OECD) guidelines for BCF prediction,^30^ *in vitro* clearance appears to have little impact on BCF values for ionizable, less lipophilic APIs.^87^ In contrast, numerous studies have proposed a significant interplay between biotransformation (metabolism) and bioaccumulation potential in nonpolar and weakly ionizable aromatic compounds.^88,89^ There is hence a need to further validate existing guidelines to better accommodate ionizable compounds, including considerations of their nonspecific binding in fish plasma.^90^

In contrast to the BCF approach, which assumes that the compound is primarily taken up from the water, elimination half-life (t_1/2_) can serve as an alternative bioaccumulation metric when other uptake or exposure routes, such as dietary uptake, are implicated.^91^ Whereas a long t_1/2_ is desirable for convenience in pharmaceutical dosing regimens in human patients, this could enhance an API’s potential to accumulate in biota in the environment, increasing the likelihood of toxicity and transfer through the food chain (biomagnification). Evidence suggests, however, that dietary uptake in fish is low compared to branchial uptake for a range of pharmaceuticals.^92,93^ Nonetheless, metabolic rate constants obtained from *in vitro* clearance assays, combined with predicted excretion rate constants, can be used to estimate APIs’ t_1/2_. Typical hepatocyte suspension assays covering a t_1/2_ of 1–2 h have been suggested to be sufficient in this regard,^94^ while monolayers and 3D cell cultures may be preferred for studying the clearance of APIs with longer t_1/2_s. However, since direct comparison of *in vitro*-derived t_1/2_ with *in vivo* data is not feasible in fish, validating these predictions remains challenging and warrants further investigation.

#### Assessing Enzyme Induction and Inhibition

In humans, the effects (i.e., induction and inhibition) of API exposure on the activity of important metabolic enzymes are particularly important for predicting and identifying drug–drug interactions. This is because altering the activity of a principal metabolic enzyme will inevitably affect the biotransformation of concurrently administered drugs. Understanding how chemical exposure affects enzyme activity in fish can help infer impacts on endogenous metabolism and – assuming comparable enzyme systems to mammals – can support the bioaccumulation assessment of both individual APIs and environmentally relevant mixtures. Equally, changes in enzyme activity can be of ecotoxicological significance due to the involvement of enzymes in the production of reactive and/or toxic metabolites and byproducts.^95^ Such data can be obtained with relative ease from *in vitro* fish liver systems. While enzyme inhibition studies are feasible in all the currently available models, liver subcellular fractions represent the most widely used and practical model systems for this application.^96,97^ Enzyme induction, on the other hand, can only be assessed in cultured cells. Studies by the coauthors of this review on the induction of CYP1A-related (i.e., 7-ethoxy-resorufin-O-deethylase, EROD) activity by a subset of common APIs in rainbow trout primary hepatocyte monolayers and static spheroid cultures have yielded complementary findings. Exposures to high concentrations of clozapine and quetiapine significantly induced EROD activity in the monolayers (following 8 h, at 100 μM) and spheroid cultures (following 72 h, at 10 μM), while propranolol exposure had no significant effect in either of the models.^64,74^ In addition, inhibition studies using rainbow trout liver S9 fractions found both clozapine and quetiapine to be moderate to weak inhibitors of EROD activity, indicating that these two compounds are likely fish CYP1A substrates, despite being noninhibitory toward human CYP1A.^96^ Taken together, these results suggest that clozapine and quetiapine can act as both inducers and inhibitors (or substrates) of CYP1A in fish.

#### Identifying Metabolites and Key Metabolic Pathways

As part of API clearance assays in liver subcellular fractions, metabolic pathways can be assessed (a.k.a. reaction phenotyping) by omitting the cofactor(s) required for specific enzyme activities (Supporting Information, Table S3) and then comparing the resulting API clearance to that obtained when all cofactors are present. For example, by only omitting NADPH – a crucial cofactor for CYP activity – the contribution of CYP enzymes to API clearance can be determined. Using this approach, the clearances of multiple APIs from various therapeutic classes have been shown to be largely dependent on CYP-mediated metabolism in rainbow trout S9 fractions,^87,97^ indicating that the CYP system may be as important for pharmaceutical metabolism in fish as it is in humans. Alternatively, the metabolic profile of an API can be investigated through a more detailed mass spectrometric analysis of incubated samples (from either cellular or subcellular test systems) to measure not only the depletion of parent compound, but also to identify and quantify metabolites, which can then be linked to potential metabolic pathways (e.g., glucuronides to UGT). Such data can provide insight into key metabolic differences between fish and mammals, as well as between different fish species. Suspension cultures of rainbow trout and common carp primary hepatocytes, for example, have been shown to accurately predict species-specific metabolite patterns of the pesticide methoxychlor in farmed fish.^50^ The authors’ own data also suggest differences in the metabolic pathways of specific APIs between fish species based on reaction phenotyping data.^75^

#### Assessing Effects on Gene and Protein Expression

Cell-based liver models, particularly monolayers and spheroids, are amenable for assessing the effects of API exposure on the expression of specific genes (transcripts). The results from such studies can help inform on mechanisms whereby the studied API(s) may alter the expression of key DMEs (thereby impacting their own metabolism or that of coadministered APIs) and other physiologically important proteins, potentially leading to endocrine disruption and/or toxicity. Exposures of rainbow trout hepatocyte monolayers to quetiapine and MPA, for example, have been shown to induce transient but statistically significant transcriptional activation of four key metabolism- and transport-related genes, namely *cytochrome P4501A* (*cyp1a*), *cytochrome P4503A* (*cyp3a*), *P-glycoprotein* (*abcb1*) and *multidrug resistance-associated protein 2* (*mrp2*).^74^ These findings suggest interaction of the APIs with the encoded enzyme and transporter proteins, as well as the potential disruption of molecular signaling pathways involving the pregnane X receptor (PXR) and aryl hydrocarbon receptor (AhR), which regulate the expression CYP3 and CYP1, respectively.^98,99^ Given that drug-metabolizing enzymes such as CYPs are well retained during primary hepatocyte isolation,^100^ liver cell cultures also provide relevant models for the targeted measurement of these (and other) cellular proteins, offering valuable insights into the post-transcriptional effects of API exposure. For example, in rainbow trout hepatocyte monolayers exposed to dexamethasone, significant reductions in CYP1A1 protein levels compared with untreated cells have been observed, evidencing responsiveness at the protein level and aligning with previous *in vivo* findings.^101^

The architecture and functionality of 2D and 3D primary hepatocyte cultures make them potentially suitable for large-scale transcriptomic, proteomic and metabolomic workflows. While such multiomics studies have yet to be applied to investigate the effects of API exposure in fish liver cell culture systems, they hold promise for providing a more comprehensive assessment of biological responses *in vitro* – both for evaluating functional integrity and for comparison with *in vivo* studies.

#### Assessing Toxicity

Various cell-based *in vitro* systems with different levels of complexity have been used to study the potential adverse effects of drugs on the human liver.^102^ Primary hepatocyte cultures can similarly be applied to assess the potential toxicity of APIs and their metabolites in fish, generating data that can be especially useful in the realms of aquaculture and ecotoxicology. While initial efforts^103^ and standardized guidelines for fish cell line acute toxicity testing (OECD Test Guideline 249)^104^ have relied on gill-derived cell lines, liver-derived cells from species such as rainbow trout,^37^ topminnow (*Poeciliopsis lucida*)^105^ and zebrafish (*Danio rerio*)^106,107^ have also been identified as promising alternatives to *in vivo* fish toxicity testing, especially when employed in 3D culture formats.^65^ Primary hepatocyte spheroid cultures are likely the most effective models for toxicity assessment as they enable the evaluation of both reduced toxicity due to metabolic clearance and elevated toxicity due to the formation of reactive or otherwise toxic metabolites. While fish liver cell lines can also be applied to assess hepatotoxicity, these systems have a limited ability to predict metabolite-induced toxicity. Previous literature has demonstrated that metabolic conversion occurring in primary rainbow trout hepatocyte cultures, but not in a fish liver cell line, can increase the toxicity of compounds such as ketoconazole,^37^ likely due to the formation of toxic metabolites as observed in mammals.^108^ Conversely, metabolism yielding inactive metabolites can reduce the toxicity of rapidly cleared pharmaceuticals, as demonstrated for propranolol, in primary hepatocytes, relative to the fish liver cell line.^37^ Additionally, 3D spheroid culture systems offer the benefit of prolonged exposure times (several days to weeks), thereby enabling the study of chronic toxicity and effects of repeated exposure.

## Final Analyses, Knowledge Gaps, and Future Perspectives

Currently available *in vitro* fish liver model systems – namely liver S9 fractions, microsomes, and primary hepatocyte suspensions, monolayers and spheroids – each offer distinct beneficial features relevant for conducting various screening assays. Given their high degree of physiological relevance, these systems are likely to contribute meaningfully to pharmaceutical ERA. Although no single *in vitro* model can necessarily fully replicate *in vivo* liver function or provide a universally applicable experimental platform, existing approaches can nonetheless inform and guide *in vivo* studies, which is crucial for prioritising chemicals for definitive ERA and reducing reliance on fish testing. Rather than serving as direct replacements, these models are currently best viewed as complementary tools in the battery of available systems for chemical risk assessment, as well as for understanding basic hepatic physiology and function in fish. In the longer term, and in conjunction with other relevant *in vitro* and *in silico* tools, however, these model systems may eventually be able to replace *in vivo* tests.

Selecting the most appropriate *in vitro* system for use in ecotoxicological and ERA contexts requires careful consideration of model performance and functionality, resource demands and informative value. Based on the critical analysis performed in this study, it is reasonable to propose that the current approaches used for mammalian models during human drug discovery and development (Figure 1) can usefully inform the selection of appropriate *in vitro* fish liver models for various ecotoxicological and environmental risk assessments (Figure 4). Evaluating the different *in vitro* models available for quantifying the hepatic clearance of APIs in fish based on the aforementioned criteria, subcellular fractions offer the most cost-effective, high-throughput systems for generating expansive data sets, enabling compound prioritisation for more comprehensive studies and supporting the development of *in silico* models. Although liver microsomes and S9 fractions do not recapitulate the full functionality of intact cells, thereby introducing some uncertainty when applied in pharmaceutical ERA, these systems could serve as effective first-tier screening tools for evaluating whether an API undergoes measurable clearance in fish, identifying metabolites and key metabolic pathways, and assessing enzyme kinetics and inhibition. At a higher level of biological complexity, hepatocyte suspensions offer greater physiological relevance while maintaining the short experimental times of subcellular models. This system is particularly valuable for investigating the impact of metabolism on API clearance while also accounting for drug transport across cell membranes, albeit only over short exposure periods. Thus, hepatocyte suspensions, like subcellular fractions, have limited applicability when it comes to predicting the CL_INT_ of APIs that are more slowly metabolized. Monolayers and 3D hepatocyte cultures, although resource-intensive, provide enhanced biological fidelity and are especially suited for targeted studies requiring detailed insights into API effects (i.e., on gene expression and metabolic enzyme induction) as a result of prolonged exposure (>4 h), and for assessing the clearance of APIs with slow metabolic rates.

**Figure 4:**
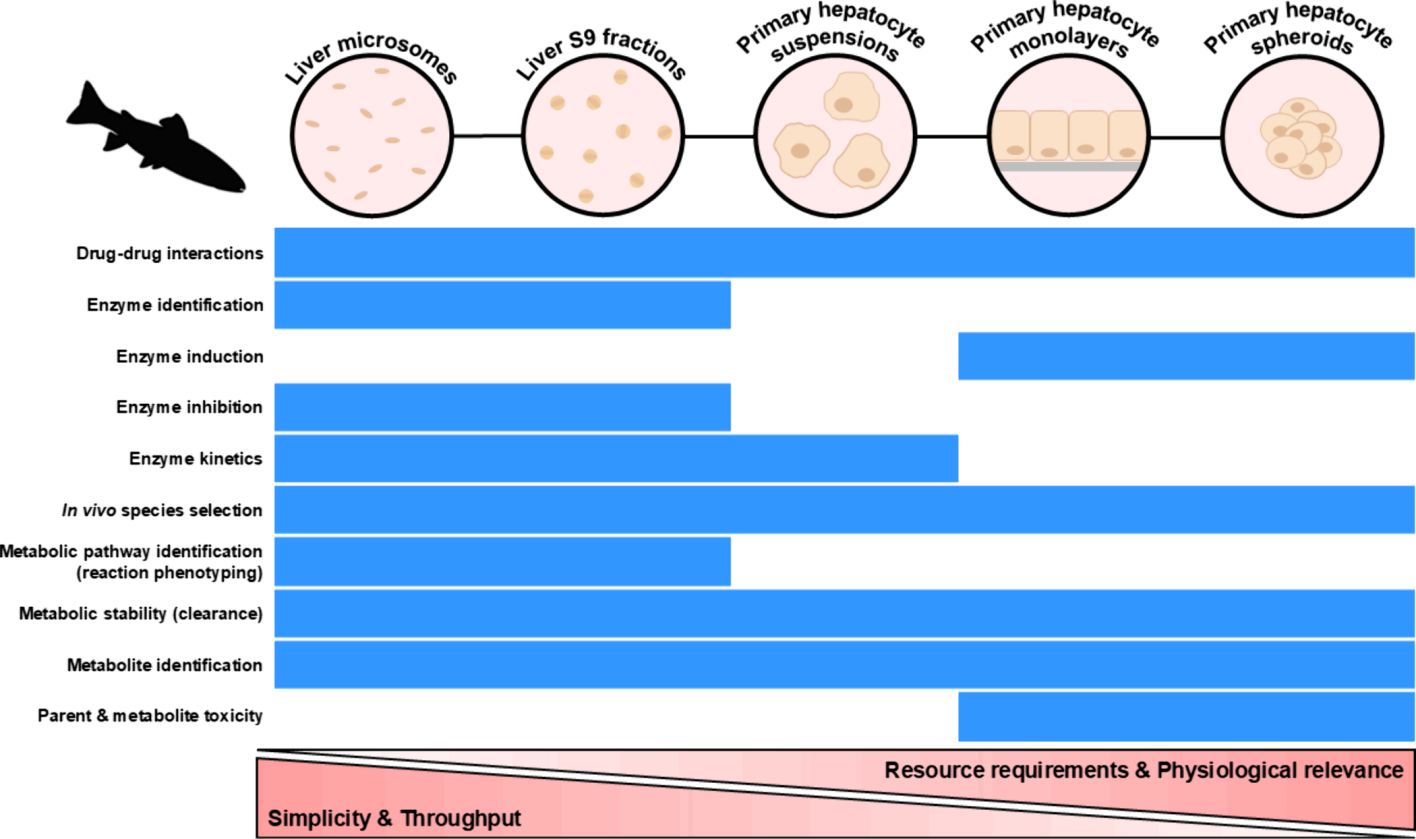
Proposed applications of *in vitro* fish liver models for ecotoxicological and environmental risk assessments. Blue and white bars indicate whether the model is proposed for the indicated purpose or not, respectively, based on practical applicability rather than theoretical possibility. Pink bars indicate (from left to right) increasing resource requirements and physiological relevance, and decreasing simplicity and throughput.

With the advent of new approach methodologies (NAMs), an increasing emphasis is being placed on the reduction, refinement and replacement of animal testing in chemical safety and risk assessment.^109^ Application of the emerging organ-on-a-chip technologies (i.e., microfluidic cell culture platforms) to study API fate and effects in fish tissues could significantly support this development. Most importantly, microfluidic platforms enable the establishment of spatiotemporal API concentration gradients to help evaluate time-dependent effect outcomes and mimic the impacts of intermittent (irregular) environmental exposures. So far, however, only a limited number of microfluidic assay platforms have been reported, utilizing either primary fish hepatocytes, or intestinal or gill cell lines.^39,40,64,75^ It is thus too early to evaluate the full potential of these technologies in pharmaceutical ERA, but preliminary studies using microfluidic primary cultures of fish hepatocyte spheroids have demonstrated comparable performance to well plate-based (static) systems in terms of API clearance and enzyme induction.^64,75^

### Knowledge Gaps and Future Perspectives

First, to facilitate the deduction of *in vitro* CL_INT_ thresholds for the identification of poorly metabolized compounds with high bioaccumulation risks in fish, more comprehensive *in vitro*-*in vivo* correlation data – including quantitative comparisons – are much needed, covering APIs from various pharmacological and physicochemical classes. Second, considering that the *in vitro* metabolic profiles and clearance of certain chemical classes have been shown to differ across fish species,^110,111^ a better understanding of species-specific pharmaceutical metabolism is required to predict differential bioaccumulation and toxicity, and inform species read-across tools. This would facilitate the identification of most-sensitive or -vulnerable species, which, in turn, could serve to inform *in vivo* studies. In theory, tools enabling read-across between mammals and fish could also support the assessment of environmental risk. However, given the substantial influence of fish-specific physiology on pharmacokinetics,^7^ such extrapolations may not currently be feasible. Fish-specific data should therefore be systematically collected in curated databases, such as the EURL ECVAM Fish *In Vitro* Intrinsic Clearance Database.^112^ Third, while *in vitro* metabolism studies often rely on single-compound exposures, this approach may overestimate compound clearance.^85^ In the environment, pharmaceuticals are typically present as part of chemical mixtures where interactions – such as the induction and inhibition of key metabolic enzymes – can alter metabolic activity. Hence, further studies on mixture toxicity and how mixtures affect metabolism and subsequent bioconcentration/bioaccumulation in fish are warranted. Indeed, efforts addressing these aims have already been reported.^77,97^ Finally, API bioavailability, which is directly linked to the potential risks a compound may pose to nontarget organisms,^113^ is not solely affected by the process of metabolism, but also by transport processes such as absorption, distribution and excretion. These latter processes are relatively understudied in fish, compared with metabolism. It is thus worthwhile further investigating relevant *in vitro* tools, such as the gill cell culture model^114,115^ and artificial permeability assay,^116^ that model chemical transport in fish. Moreover, since organs other than the liver (i.e., the intestine, gills and brain) have been shown to contribute to xenobiotic clearance in fish,^82,117–119^ the contribution of extrahepatic metabolism to total API clearance needs to be explored further. These data could be of great value for refining and validating *in silico* models, such as the fish physiologically based kinetic model,^120^ and other *in vitro-in vivo* extrapolation methods,^118,121^ thereby contributing to improved ecotoxicological screening and risk assessment approaches.

## Supplementary Material



## Notes

Experimental details and meta-data relating to Figure 3 (including Table S1 and Table S2); Main phase I and II enzymes involved in xenobiotic metabolism, together with their respective cofactors, cellular distributions and metabolic reactions (Table S3); Strengths and limitations of currently available *in vitro* fish liver models (Table S4); Average basal metabolic enzyme activity levels in different rainbow trout liver subcellular and whole-cell systems (Table S5); Resource considerations for active pharmaceutical ingredient (API) clearance assays using different *in vitro* fish liver models (Table S6) (PDF)

## ABBREVIATIONS

3D: three-dimensional
API: active pharmaceutical ingredient
BCF: bioconcentration factor
CL_INT_: intrinsic clearance
CYP: cytochrome P450
DME: drug-metabolizing enzyme
ERA: environmental risk assessment
EROD: 7-ethoxy-resorufin-O-deethylase
K_OW_: *n*-octanol–water partition coefficient
MPA: mycophenolic acid
OECD: Organisation for Economic Cooperation and Development
t_1/2_: elimination half-life
UGT: uridine 5′-diphospho-glucuronosyltransferase
