# Supplementary material for: In Vitro Liver Models for Studying Pharmaceutical Metabolism in Fish – A Critical Analysis of Their Applications and Limitations
Source: Environ Sci Technol. 2026 May 27;60(22):15435–48. doi: 10.1021/acs.est.6c00280 (PMC13262058; doi:10.1021/acs.est.6c00280)
Supplement: Supplementary file 1 [file es6c00280_si_001.pdf]

Supporting Information

In Vitro Liver Models for Studying Pharmaceutical Metabolism in Fish – A Critical Analysis on Their Applications and Limitations

Chrisna Matthee[,1], Tea L. M. Pihlaja[2], Päivi Järvinen[2], A. Ross Brown[1], Anke Lange[1], Tiina M. Sikanen[2,3] and Charles R. Tyler*[,1]*

[1]Faculty of Health and Life Sciences, Biosciences, University of Exeter, Exeter, EX4 4QD, United Kingdom

[2]Faculty of Pharmacy, Drug Research Program, University of Helsinki, Helsinki, FI-00790, Finland

[3]Helsinki Institute of Sustainability Science, University of Helsinki, Helsinki, FI-00100, Finland

Summary of Supporting Information

- Number of pages: 14
- Number of tables: 6 (Table S1-S6)
- Number of figures: 1

TABLE OF CONTENTS

Table of Contents ……….S1

Experimental details and meta-data relating to Figure 3 ……….S2

Table S1. The liquid chromatographic method used for the quantitation of olaparib in rainbow trout hepatocyte incubations.

Figure S1. Log_{10}-linear regressions of olaparib depletion in primary rainbow trout hepatocyte suspensions and spheroids.

Table S2. *In vitro* intrinsic clearances of the model pharmaceuticals determined using the substrate depletion approach in different rainbow trout *in vitro* matrices.

Table S3. Main phase I and II enzymes involved in xenobiotic metabolism, together with their respective co-factors, cellular distributions and metabolic reactions. ……….S5

Table S4. Strengths and limitations of currently available *in vitro* fish liver models. ……….S6

Table S5. Average basal metabolic enzyme activity levels in different rainbow trout liver subcellular and whole-cell systems. ……….S8

Table S6. Resource considerations for active pharmaceutical ingredient (API) clearance assays using different *in vitro* fish liver models. ..……...S10

References ..……...S12

Experimental details and meta-data relating to Figure 3

Experimental details for unpublished data

The experimental data relating to the extrapolated *in vivo* intrinsic clearance (CL_{INT}) rates of olaparib in primary rainbow trout hepatocyte suspensions (SUSP) and static primary hepatocyte spheroid cultures (SS), as shown on **Figure 3** of the main manuscript, were obtained as described previously, [1] using cryopreserved primary hepatocytes of rainbow trout purchased from Primacyt Cell Culture Technology GmbH (#RTH180219, pool of six sexually immature female fish).

The *in vitro* elimination rate constant in SUSP was determined in accordance with OECD 319A using a single-vial method. The substrate depletion assays were carried out at 11 ± 1 °C, using freshly thawed cells (1×10^6 cells/mL) and 1 µM olaparib (including 0.5 %, v/v, residual DMSO) in a total volume of 0.5 mL of L-15 medium (Leibovitz). Samples were taken at eight different timepoints between 20 and 240 min. The *in vitro* elimination rate constant in SS was determined using spheroids cultured on AggreWell™400 plates (Stemcell Technologies; initial seeding density of 0.06×10^6 cells/mL) for 8 days, after which the spheroids (ca. 1200 per well) were collected, separated from the culture medium by centrifugation, and resuspended in 100 µL of basal L-15 medium containing 1 µM olaparib (including 0.5 %, v/v, residual DMSO). In this case, the substrate depletion rate was measured separately (with a batch of ca. 1200 spheroids) at each incubation time (30-240 min).

To determine the remaining olaparib concentration at each incubation time, the samples were analysed using the liquid chromatography method described in **Table S1**. The substrate depletion rate (h^{-1}) was defined as the slope of the $\log_{10}$-linear regression of the remaining olaparib concentration in the sample vs. incubation time, using an average of two independent incubation series of active and inactive hepatocytes (**Figure S1**). *In vitro* clearance was calculated as described previously [1] and extrapolated to *in vivo* intrinsic clearance using a scaling factor of 510×10^6 cells/g liver.

Table S1. The liquid chromatographic method used for the quantitation of olaparib in rainbow trout hepatocyte incubations.

**Instrument:** Agilent 1200 with DAD (G1315C) and fluorescence (G1321A) detectors		
**Separation column:** Agilent Poroshell 120 EC-C18 (4.6 x 50mm, 2.7 µm)		
**Eluents:** A = 0.1% phosphoric acid (aqueous), B= 0.1% phosphoric acid in acetonitrile		
**Compound**	**Olaparib**	
**Solvent gradient**	**Time (min)**	**%B**
	0.00	15
	1.00	15
	4.00	45
	4.05	90
	4.50	90
	4.60	15
**t_R (min)**	3.35	
**$V_{injection}$ (µL)**	5	
**Detection**	206 nm	
**Linearity**	y = 0.0067x + 0.0760	
**R^2 (range)**	0.9994 (0.08 - 0.5 µM)	

The method was validated with respect to selectivity, linearity, range, precision, accuracy, and recovery, adhering to criteria set in the U.S. FDA's Bioanalytical Method Validation guidance. [2]

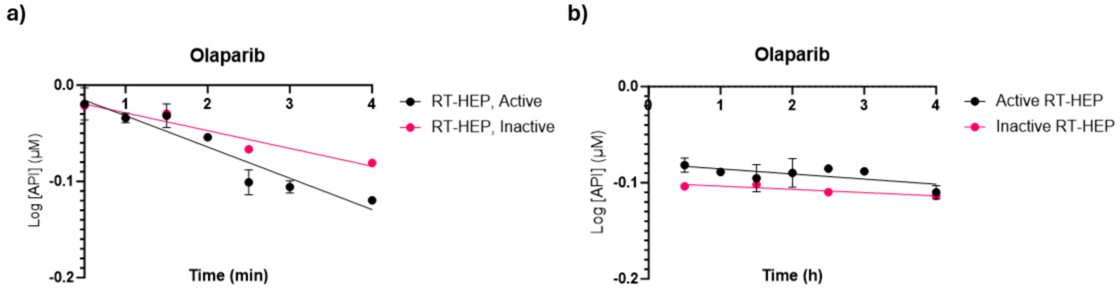

Figure S1. Log_{10}-linear regressions of olaparib depletion in (a) primary rainbow trout hepatocyte suspensions (0.047 mL/h/10^6 cells; R^2 = 0.87) and (b) primary rainbow trout hepatocyte spheroids (no statistically significant clearance). The rate of abiotic loss detected in inactivated hepatocytes was subtracted from the clearance rate detected in active hepatocytes.

Meta-data relating to Figure 3

Table S2. *In vitro* intrinsic clearances of the model pharmaceuticals determined using the substrate depletion approach in different rainbow trout *in vitro* matrices.

API	S9			SUSP			ML			SS		
	CL_{INT} (mL/h/g liver)	SEM	N	CL_{INT} (mL/h/g liver)	SEM	N	CL_{INT} (mL/h/g liver)	SEM	N	CL_{INT} (mL/h/g liver)	SEM	N
Propranolol	160.0	4.3	2	230.2	8.2	1	79.6	14.1	2	257.0	172.9	1
Quetiapine	22.0	4.1	2	112.0	11.0	1	49.7	5.9	3	nd		
MPA	89.0	4.2	2	36.2	5.4	1	64.0	12.4	3	171.3	115.9	1
Clozapine	nd			nd			11.6	4.8	4	nd		
Olaparib	nd			24.0	12.0	1	3.8	2.7	2	nd		

Abbreviations: API, active pharmaceutical ingredient; CL_{INT}, extrapolated in vivo intrinsic clearance; ML, primary hepatocyte monolayer cultures; MPA, mycophenolic acid; N, biological replicates; nd, clearance not detectable; S9, liver S9 fractions; SEM, standard error of the mean; SS, static primary hepatocyte spheroid cultures; SUSP, primary hepatocyte suspension cultures.

Table S3. Main phase I and II enzymes involved in xenobiotic metabolism, together with their respective co-factors, cellular distributions and metabolic reactions.

	Enzyme Class	Co-Factors	Cellular Distribution	Metabolic Reactions
Phase I	Alcohol dehydrogenase (ADH)	NAD^+	Cytosol	Alcohol oxidation
	Aldehyde dehydrogenase (ALDH)	NAD^+	Cytosol, mitochondria	Aldehyde oxidation
	Aldehyde oxidase (AO)	FAD, MoCo	Cytosol	Aldehyde oxidation; N-heterocycle hydroxylation
	Carboxylesterase (CES)	-	Mainly ER (luminal surface), cytosol	Ester, amide, carbamate & thioester hydrolysis
	Cytochrome P450 (CYP)	NADPH	ER (cytosolic surface)	Oxidation, hydroxylation, dealkylation
	Flavin-containing monooxygenase (FMO)	FAD, NADPH	ER (cytosolic surface)	Oxidation
	Monoamine oxidase (MAO)	FAD	Mitochondria (cytosolic surface)	Oxidative deamination
Phase II	Glutathione S-transferase (GST)	GSH	Cytosol (mainly)	Glutathione conjugation
	Methyl transferase (MET)	SAM	Cytosol, ER (cytosolic surface)	Methylation
	N-acetyl transferase (NAT)	Acetyl Coenzyme A	Cytosol	Acetylation
	Sulfotransferase (SULT)	PAPS	Cytosol	Sulfation
	UDP-glucuronosyltransferase (UGT)	UDPGA	ER (luminal surface)	Glucuronidation

The marker reactions used to assess specific enzyme activity are typically those recommended in the OECD 319A/B guidance documents, [3,4] or those employed during human drug interaction assessment. [5]

Abbreviations: ER, endoplasmic reticulum; FAD, flavin adenine dinucleotide; GSH, glutathione; MoCo, molybdenum cofactor; NAD^+, nicotinamide adenine dinucleotide; NADPH, reduced nicotinamide adenine dinucleotide phosphate; PAPS, 3’-phosphoadenosine-5’-phosphosulfate; SAM, S-adenosyl-L-methionine; UDPGA, uridine diphosphate glucuronic acid. Adapted from literature. [1-9]

Table S4. Strengths and limitations of currently available *in vitro* fish liver models.

System	Strengths	Limitations
Common to all (except for cell lines)	Can use single or pooled donor fish.<br>Can prepare from fresh or cryopreserved sources.<br>Allow study of inter-individual variability.<br>Allow identification of metabolites.	Animal sacrifice required.
Liver subcellular fractions (microsomes and S9 fractions)	Amenable to higher-throughput screening and automation.<br>Relatively low cost.<br>Ease of use and storage.<br>Short assay duration.<br>Most phase I & II metabolic enzymes present.<br>Allow identification of key metabolic pathways.	Additional co-factors required.<br>Potential inactivation of enzymes during preparation.<br>Lack of cellular compartmentalisation.<br>Restricted assay duration (hours).<br>Not suitable for studying gene or protein expression.<br>Chemical transport over cell membranes not accounted for.
Primary hepatocytes cultures (suspensions and monolayers)	Intact cell membranes, organelles & membrane transporters.<br>All metabolic enzymes & co-factors present at physiological concentrations.<br>No additional co-factors required.<br>Relatively long viability (several days; monolayers), allowing assessment of slowly metabolised compounds.<br>Chemical efflux, uptake and diffusion over the plasma membrane are accounted for.<br>Re-establishment of cell-cell contacts (monolayers).<br>Suitable for studying gene and protein expression.	Restricted assay duration (hours; suspensions).<br>Lack of cell-cell interactions (suspensions).<br>Relatively time-consuming & costly (monolayers > suspensions).<br>Static incubation conditions (monolayers).<br>Some differentiated cell features lost during long-term culture (monolayers).<br>Special culture vessels with surface coatings required (monolayers).<br>Loss of metabolic activity over time.<br>Potential contamination from non-applicable cell types can compromise model integrity.<br>Non-homeostatic cell culture conditions – impaired cell differentiation.<br>Lack cellular heterogeneity and density of the liver.

Table S4. Strengths and limitations of commonly used *in vitro* fish liver models. (continued)

System	Strengths	Limitations
Primary hepatocyte spheroid cultures	Relatively high physiological relevance – increased cell density, cell-cell signalling, etc.<br>Superior maintenance of differentiated hepatic phenotype.<br>Superior maintenance of cell viability (weeks).<br>Superior metabolic activity (often).<br>Suitable for studying gene and protein expression.<br>Gene expression closely resembles that *in vivo* (later culture).<br>Allow assessment of slowly metabolised compounds.	High level of variability between cultures, resulting in inconsistent phenotypic traits.<br>Variable chemical exposure of cells within the spheroid structure.<br>Relatively labour-intensive, time-consuming and costly.<br>Special culture vessels and labware required.<br>Low applicability for high-throughput screening.<br>Lack of reliable protocols.<br>Lack cellular heterogeneity and density of the liver.
Immortalised liver cell lines (monolayer and spheroid cultures)	Relatively low cost.<br>Ease of use.<br>No animal sacrifice required.	Dedifferentiated phenotype.

Adapted from literature; [6] supplemented with original insights.

Table S5. Average basal metabolic enzyme activity levels in different rainbow trout liver subcellular and whole-cell systems.

	Liver S9 Fractions	Liver Microsomes	Primary Hepatocyte Cultures		
			Suspensions	Monolayers	Spheroids*
**CYP1A**	2.25 ± 0.35 (phenacetin) [a]<br>0.65 ± 0.41 (7-ER) [b]<br>~1.0 (7-ER) [k]<br>3.89 ± 0.29 (7-ER) [m]<br>10.68 ± 3.67 (7-ER) [n]<br>4.6 ± 0.1 (7-ER) [o]	2.44 ± 1.51 (7-ER) [b]<br>2.58 ± 0.22 nM/min (7-ER) [c]	0.2 ± 0.1 (7-ER) [d]<br>0.96 ± 0.33 (7-ER) [e]<br>3.0 ± 1.9 (7-ER) [h]<br>6.2 ± 1.1 (7-ER) [i]<br>7.9 ± 3.2 (7-ER) [j]<br>2.74 ± 1.21 (7-ER) [l]	1.96 ± 0.55 (7-ER) [e]<br>~2.0 (7-ER) [f]<br>1.38 ± 0.31 (7-ER) [g]	1.29 ± 0.48 (7-ER; d 5) [e]<br>1.03 ± 0.65 (7-ER; d 30) [e]<br>~1.2 (7-ER; d 5) [f]<br>~1.0 (7-ER; d 30) [f]<br>4.9 ± 2.1 (7-ER; d 9) [h]
**CYP3A**	1.78 ± 0.91 (midazolam) [a]<br>36.6 ± 6.0 (testosterone) [b]<br>~16.2 (BFC) [k]	71.2 ± 7.6 (testosterone) [b]<br>56.7 ± 12.7 nM/min (BFC) [c]	33.2 ± 8.9 (testosterone) [l]		
**UGT**	398.5 ± 64.5 nM/min [a]<br>9150 ± 0.91 (pNP) [b]<br>~416.2 (pNP) [k]<br>1014 ± 44.9 (pNP) [m]<br>3150 ± 391 (pNP) [n]<br>923 ± 101 (pNP) [o]	24,700 ± 9.1 (pNP) [b]	30.4 ± 16.0 [e]<br>390 ± 190 (pNP) [i]<br>230 ± 40 (pNP) [j]	55.2 ± 20.8 [e]<br>~50.0 (pNP) [f]	34.8 ± 23.0 (d 5) [e]<br>66.8 ± 5.8 (d 30) [e]<br>~40.0 (pNP; day 5) [f]<br>~70.0 (pNP; d 30) [f]<br>~20 (8-HQG; d 9) [h]

The marker reactions used to assess specific enzyme activity are typically those recommended in the OECD 319A/B guidance documents, [3,4] or those employed during human drug interaction assessment. [5] Enzymes activities are reported in pmol/min/mg protein, unless otherwise indicated, with substrates and culture duration (where known and applicable) shown in parentheses. Blank cells indicate the absence of published data. *Only enzyme activities for spheroids established using primary hepatocytes are presented.

[a]Enzyme activities were determined using pooled subcellular fraction at 1 mg/mL protein in 0.1 M phosphate buffer at 14 °C for 15 min (CYP) or 30 min (UGT and SULT) using a cocktail assay of known (human) marker activities of the respective enzymes. [7]

[b]Enzyme activities were determined at 0.25 mg/mL protein (S9 & microsomes; CYP1A), 1 mg/mL protein (microsomes; CYP3A & UGT) or 2 mg/mL protein (S9; CYP3A & UGT) in Hank's balanced salt solution with calcium at 10 °C using pooled subcellular fraction from male fish (~1.5 y of age). [8]

[c]Enzyme activities were determined at 0.5 mg/mL protein in 0.1 M potassium phosphate buffer at 21±1 °C for 10 min (CYP1A) or 20 min (CYP3A) using pooled microsomes from male fish. [9]

[d]Enzyme activities were determined after 24 hrs in culture using non-pooled hepatocytes from 9 individual diploid female fish (~350 g). [10]

[e]Conditions and some substrates not specified; hepatocytes isolated from male fish (700-800 g). [11]

[f]Conditions not specified; hepatocytes isolated from 3-5 individual male fish (700-800 g). [12]

[g]Enzyme activity was determined in 50 mM phosphate buffer (pH 7.8) at room temperature using pooled hepatocytes from 3 triploid female fish (80-200 g). [13]

[h]Enzyme activities were determined at $3x10^5$ cells/mL at 15 °C using pooled, cryopreserved hepatocytes from 6 sexually immature female fish. [1]

[i]Enzyme activities were determined at $1x10^6$ cells/mL for 30 min (CYP1A) or at $1x10^7$ cells/mL for 60 min (UGT) in 100 mM phosphate buffer at 11 °C using non-pooled, freshly isolated hepatocytes from 3-5 individual male fish (~500 g). [14]

[j]Enzyme activities were determined at $1x10^6$ cells/mL for 30 min (CYP1A) or at 10x106 cells/mL for 60 min (UGT) in 100 mM phosphate buffer at 11 °C using non-pooled, freshly isolated hepatocytes from 3-5 individual female fish (~500 g). [14]

[k]Enzyme activities were determined at 1 mg/mL protein in 100 mM phosphate buffer (pH 7.8) using pooled S9 fractions from 4 fish, and represent the maximum reaction rate. [15]

[l]Enzyme activities were determined in Hank's balanced salt solution with calcium (pH 7.8) at 10 °C for up to 40 min using non-pooled, freshly isolated hepatocytes from 5 individual male fish (~1.5 y of age). [16]

[m]Enzyme activities were determined at 11 °C, pH 7.8 using liver S9 fractions pooled from male and female fish (~400-700 g). A pooled mean + SD was calculated across 6-12 replicates. [17]

[n]Enzyme activities were determined using liver S9 fractions pooled from male and female fish (~4-400 g) at 1 mg/mL protein in phosphate buffer (11 °C, pH 7.8). A pooled mean + SD was calculated across 16 replicates. [18]

[o]Enzyme activities were determined using liver S9 fractions pooled from male and female fish (~380 g) at 1 mg/mL protein in phosphate buffer (11 °C, pH 7.8). [19]

Abbreviations: 7-ER, 7-ethoxyresorufin; 8-HQG, 8-hydroxyquinoline glucuronidase; BFC, benzyloxy-4-trifluoromethylcoumarin; CYP, cytochrome P450; d, day; pNP, p-nitrophenol; SULT, sulfotransferase; UGT, uridine 5'-diphospho-glucuronosyltransferase.

Table S6. Resource considerations for active pharmaceutical ingredient (API) clearance assays using different *in vitro* fish liver models.

Considerations	Common to All	Liver Subcellular Fractions	Primary Hepatocyte Cultures<br>Suspensions	Monolayers	Spheroids
Cost (per test substance)	Solvents<br>APIs<br>Buffer salts<br>Analytical chemistry	**Estimated at ~40 € including:**<br>• Commercial S9 fractions [a,b]<br>• Co-factors [b]<br>• Pore-forming agent [b]	**Estimated at ~600 € including:**<br>• Commercial cryopreserved cells and thawing kits [a,c]<br>• Cell incubation medium	**Estimated at ~625 € including:**<br>• Commercial cryopreserved cells and thawing kits [a,d].<br>• Cell culture medium<br>• Cell culture plates [d]<br>• Membrane matrix (plate coating).<br>• Media supplements	**Estimated at ~ 650-700 € including:**<br>• Commercial cryopreserved cells and thawing kits [a,d].<br>• Cell culture medium<br>• Cell culture plates [d]<br>• Anti-adherence solution (plate coating).<br>• Media supplements
Time	Fish sourcing, transport & acclimatisation [a].<br>Hepatocyte isolation [a].<br>Sample preparation.<br>Analytical method development & validation (several days/substance).<br>Analytical chemistry (~1 day/substance).	Subcellular fraction preparation [a].<br>API exposure (≤ 4 hrs per experiment).	API exposure (≤ 4 hrs per experiment).<br>Cell counts & viability estimations.	Plate coating.<br>Cell culture prior to API exposure (1-2 d).<br>API exposure (up to 24 hrs or more per experiment).<br>Cell counts & viability estimations.<br>Microscopic examination and/or imaging.	Cell culture prior to API exposure (≥ one week).<br>API exposure (up to 24 hrs or more per experiment).<br>Cell counts & viability estimations.<br>Microscopic examination and/or imaging.

Cost estimates are based on commercially available liver S9 fractions or cryopreserved primary hepatocytes, which are available at an average price of ~100 € per 10 mg S9 protein (one vial) or ~500 € per 2 million cells, respectively. [20]

[a]If performing in-house hepatocyte isolations, the required resources include fish (including transport and husbandry) and the costs associated with the hepatocyte isolation procedure.

[b]Assuming a total consumption of 2 mg S9 protein (~20 €) per clearance determination of one test substance, including two technical replicates each of active and inactivated S9 fractions, as advised in the standardised test protocol. For example, by using 1 mg/mL total S9 protein concentration in 0.5 mL incubation volume, enabling sampling at 9-10 different

timepoints; and an additional cost of ~20 € (including technical replicates) associated with the necessary cofactors (assuming 2 mM NADPH, 2 mM UDPGA, 0.1 mM PAPS, and 5 mM glutathione concentrations), as well as alamethicin (25 µg/mL; pore-forming agent added to overcome UGT latency), excluding the cost of the incubation buffer.

[c]Assuming a total consumption of 2 million cells (one vial, ~500 €) per clearance determination of one test substance, including two technical replicates each of active and inactivated cells, as advised in the standardised test protocol. For example, by using 1 million cells/mL cell density in 0.5 mL incubation volume, enabling sampling at 9-10 different time points; and an additional cost of ~100 € associated with the (commercial) thawing kit and the incubation medium.

[d]Assuming approximately the same total consumption of cells (one vial, ~500 €) per clearance determination of one test substance, including two technical replicates each of active and inactivated cells, similar to the suspension assays. For example, by using an initial seeding density of 0.6 million cells/well (monolayer cultures on 24-well plates and 9-10 samplings per well) or 0.06 million cells/well (spheroid cultures on 24-well AggreWell plates and 9-10 wells of spheroids used for the clearance assays); and an additional cost of ~100 € associated with the (commercial) thawing kit and incubation medium, as well as an additional cost of ~25 € (monolayer cultures) and up to 100 € (spheroid cultures) associated with the specialised well plates and coatings needed for cell culturing.

REFERENCES

(1) Prioritisation and Risk Evaluation of Medicines in the EnviRonment (PREMIER). *Evaluation of fish gill uptake and hepatic clearance for 10-20 APIs, including feasibility of the OECD 319b test or organ-chips for prediction of in vivo hepatic clearance of APIs in fish.* Internal report (D2.10). Work package 2.3: *In vitro* effects models, 2024.

(2) U.S. Food and Drug Administration. *Bioanalytical method validation: Guidance for industry*. 2018. www.fda.gov/files/drugs/published/Bioanalytical-Method-Validation-Guidance-for-Industry.pdf (accessed March 2026).

(3) Organisation for Economic Cooperation and Development (OECD). Determination of *in vitro* intrinsic clearance using cryopreserved rainbow trout hepatocytes (RT-HEP) (Test No. 319A). In *OECD Guidelines for the Testing of Chemicals, Section 3*, 2018.

(4) Organisation for Economic Cooperation and Development (OECD). Determination of *in vitro* intrinsic clearance using rainbow trout liver S9 sub-cellular fraction (RT-S9) (Test No. 319B). In *OECD Guidelines for the Testing of Chemicals, Section 3*, 2018.

(5) International Council for Harmonisation of Technical Requirements for Pharmaceuticals for Human Use. ICH harmonised guideline: Drug interaction studies M12. https://database.ich.org/sites/default/files/ICH_M12_Step4_Guideline_2024_0521_0.pdf: 2024.

(6) Collins, S. D.; Yuen, G.; Tu, T.; Budzinska, M. A.; Spring, K.; Bryant, K.; Shackel, N. A. *In vitro* models of the liver: disease modeling, drug discovery and clinical applications. In *Hepatocellular Carcinoma*, Tirnitz-Parker, J. E. E. Ed.; Codon Publications, 2019; pp 47-67.

(7) PRIMACYT Cell Culture Technology GmbH. Germany.

(8) Han, X.; Nabb, D. L.; Yang, C.; Snajdr, S. I.; Mingoia, R. T. Liver microsomes and S9 from rainbow trout (*Oncorhynchus mykiss*): comparison of basal-level enzyme activities with rat and determination of xenobiotic intrinsic clearance in support of bioaccumulation assessment. *Environmental Toxicology and Chemistry* **2009**, *28* (3), 481-488. DOI: 10.1897/08-269.1.

(9) Pihlaja, T. L. M.; Niemissalo, S. M.; Sikanen, T. M. Cytochrome P450 inhibition by antimicrobials and their mixtures in rainbow trout liver microsomes *in vitro*. *Environmental Toxicology and Chemistry* **2022**, *41* (3), 663-676. DOI: 10.1002/etc.5160.

(10) Bartram, A. E.; Winter, M. J.; Huggett, D. B.; McCormack, P.; Constsantine, L. A.; Hetheridge, M. J.; Hutchinson, T. H.; Kinter, L. B.; Ericson, J. F.; Sumpter, J. P.; Owen, S. F. *In vivo* and *in vitro* liver and gill EROD activity in rainbow trout (*Oncorhynchus mykiss*) exposed to the beta-blocker propranolol. *Environmental Toxicology* **2012**, *27* (10), 573-582. DOI: 10.1002/tox.20684.

(11) Flouriot, G.; Monod, G.; Valotaire, Y.; Devaux, A.; Cravedi, J. Xenobiotic metabolizing enzyme activities in aggregate culture of rainbow trout hepatocytes. *Marine Environmental Research* **1995**, *39*, 293-297. DOI: 10.1016/0141-1136(94)00027-M.

(12) Cravedi, J. P.; Paris, A.; Monod, G.; Devaux, A.; Flouriot, G.; Valotaire, Y. Maintenance of cytochrome P450 content and phase I and phase II enzyme activities in trout hepatocytes cultured as spheroidal aggregates. *Comparative Biochemistry and Physiology* **1996**, *113* (2), 241-246. DOI: 10.1016/0742-8413(95)02093-4.

(13) Matthee, C.; Brown, A. R.; Lange, A.; Trznadel, M.; Tyler, C. R. Metabolic clearance of physicochemically diverse pharmaceuticals in cultured fish hepatocytes. *Environmental Toxicology and Chemistry* **2025**, *44* (11), 3271-3281. DOI: 10.1093/etojnl/vgaf193.

(14) Fay, K. A.; Fitzsimmons, P. N.; Hoffman, A. D.; Nichols, J. W. Optimizing the use of rainbow trout hepatocytes for bioaccumulation assessments with fish. *Xenobiotica* **2014**, *44* (4), 345-351. DOI: 10.3109/00498254.2013.845704.

(15) Martin, B. P. M.; Franco, M. E.; Schirmer, K. Comparative characterization of organ-specific phase I and II biotransformation enzyme kinetics in salmonid S9 sub-cellular fractions and cell lines. *Cell Biology and Toxicology* **2025**, *41*. DOI: 10.1007/s10565-025-09992-8.

(16) Nabb, D. L.; Mingoia, R. T.; Yang, C.; Han, X. Comparison of basal level metabolic enzyme activities of freshly isolated hepatocytes from rainbow trout (*Oncorhynchus mykiss*) and rat. *Aquatic Toxicology* **2006**, *80*, 52-59. DOI: 10.1016/j.aquatox.2006.07.012.

(17) Saunders, L. J.; Fitzsimmons, P. N.; Nichols, J. W.; Gobas, F. A. P. C. *In vitro-in vivo* extrapolation of hepatic and gastrointestinal biotransformation rates of hydrophobic chemicals in rainbow trout. *Aquatic Toxicology* **2020**, *228*. DOI: 10.1016/j.aquatox.2020.105629.

(18) Fitzsimmons, P. N.; Hoffman, A. D.; Fay, K. A.; Nichols, J. W. Allometric scaling of hepatic biotransformation in rainbow trout. *Comparative Biochemistry and Physiology Part C* **2018**, *214*, 53-60. DOI: https://doi.org/10.1016/j.cbpc.2018.08.004.

(19) Ladd, M. A.; Fitzsimmons, P. N.; Nichols, J. W. Optimization of a UDP-glucuronosyltransferase assay for trout liver S9 fractions: activity enhancement by alamethicin, a pore-forming peptide. *Xenobiotica* **2016**, *46* (12), 1066-1075. DOI: 10.3109/00498254.2016.1149634.

(20) PRIMACYT Cell Culture Technology. *Our Products*. https://primacyt.com/products/ (accessed 16 March 2026).
